# Rhizosphere Bacteria From *Panax notoginseng* Against *Meloidogyne hapla* by Rapid Colonization and Mediated Resistance

**DOI:** 10.3389/fmicb.2022.877082

**Published:** 2022-04-28

**Authors:** Wentao Wu, Jingjing Wang, Zhuhua Wang, Liwei Guo, Shusheng Zhu, Youyong Zhu, Yang Wang, Xiahong He

**Affiliations:** ^1^Key Laboratory of Agricultural Biodiversity and Pest Control, College of Plant Protection, Yunnan Agricultural University, Kunming, China; ^2^School of Landscape and Horticulture, Southwest Forestry University, Kunming, China

**Keywords:** rhizosphere bacteria, biological control, *Meloidogyne hapla*, rapid colonization, resistance induction

## Abstract

Root-knot nematodes (RKNs) are soil-borne pathogens that severely affect *Panax notoginseng* growth and productivity. Thus, there is an urgent need for biological control agents or green nematicides to control root-knot nematodes. Rhizosphere bacteria can effectively control RKNs through different mechanisms. In this study, the three rhizosphere *Bacillus* strains, isolated from the root of *P. notoginseng*, were evaluated for the nematicidal activity and biological control efficacy against root-knot nematodes. In addition, we also evaluated the colonization ability of the two bacterial strains with significant biocontrol effect and dynamic regulation of genes related to systemic resistance in *P. notoginseng*. The rhizosphere *Bacillus velezensis* GJ-7 and *Bacillus cereus* NS-2 showed high nematicidal activity against *Meloidogyne hapla in vitro* and significantly reduced the number of root galls in three different control experiments. The results of colonization experiments showed that the strains GJ-7 and NS-2 colonized *P. notoginseng* root rapidly and stably. Additionally, the colonization of the strains NS-2 and GJ-7 activated the defense-responsive genes in *P. notoginseng*. These results indicated that the *B. cereus* strain NS-2 and *B. velezensis* strain GJ-7 have the potential for successful ecological niche occupation and enhance plant resistance and therefore could be considered as potential biocontrol agents against root-knot nematodes.

## Introduction

Root-knotnematodes (*Meloidogyne* spp.) are obligate plant parasites that cause severe damage to the host plant, including agricultural crops, vegetables, and even medicinal plants (Anwar and McKenry, 2010; Wang et al., 2021). Plant-parasitic nematodes cause losses of more than $157 billion worldwide every year (Anwar and McKenry, 2012). Due to the short life cycle and high reproduction rates of root-knot nematodes, the control of these has been particularly challenging. Previously, chemical pesticides are effective in controlling RKNs, such as carbofuran, dazomet, fenamiphos, and sebufos (Giannakou and Karpouzas, 2003). However, due to their high virulence, they have been found to be harmful to the eco-environment, animals, and humans and have been banned in many countries (Schneider et al., 2003; Nyczepir and Thomas, 2009). Therefore, researchers are also seeking new and alternative biological control options to solve these negative impacts and the significant economic losses. In the present study, the use of microbial agents to control plant-parasitic nematodes is a potential method, such as bacteria (Stirling et al., 2017; Viljoen et al., 2019), fungi (Hussain et al., 2018, 2020), and actinomycetes (Nimnoi et al., 2017), which are nematophagous or antagonistic for root-knot nematodes. Most notably, the genus *Bacillus* can significantly reduce root-knot nematode galling and egg production and has been commercially used in many countries for the control of plant-parasitic nematodes (Oka et al., 1993; Terefe et al., 2009; Higaki and Araujo, 2012; Jamal et al., 2017; Xiang et al., 2017).

In addition, the plant rhizosphere contains a large number of beneficial bacteria, which colonize on the root surface to form biofilms so as to protect plants from disease stresses and promote plant growth (Watnick and Kolter, 2000; Nan et al., 2021). Rhizosphere bacteria can effectively control RKNs through different mechanisms. Some bacteria can produce secondary metabolites with nematicidal activity (Kerry, 2000). Other bacteria species can rapidly colonize the feeding sites of RKNs and reduce the infection of root-knot nematodes by competing for niche and nutrition (Hashem and Abo-Elyousr, 2011). Bacteria can also alter plant root exudates and induce plant resistance to reduce RKN damage (Dutta et al., 2012). Therefore, plant rhizosphere bacteria play an important role in the control of nematode disease.

Sanqi [*Panax notoginseng* (Burk.) F. H. Chen] is one of the most important Chinese medicinal plants (Yang et al., 2015). In recent years, root-knot nematodes have seriously affected the growth and productivity of *P. notoginseng* (Wang et al., 2021). In previous studies, we obtained three rhizosphere *Bacillus* isolates from *P. notoginseng*, which were resistant to root-knot nematodes. According to the amplification results of 16S rRNA sequence, the three rhizosphere strains were identified as *Bacillus cereus* NS-2, *Lysinibacillus* NS-3, and *Bacillus velezensis* GJ-7, respectively. In this study, we evaluated their nematicidal activity and biological control efficacy against root-knot nematodes. In addition, we also evaluated the colonization ability of the NS-2 and GJ-7 strains with a significant biocontrol effect and dynamic regulation of genes related to systemic resistance in *P. notoginseng.* The purpose of this study was to screen the efficient bacterial strains for the control of the root-knot nematode to *P. notoginseng* and to provide a theoretical basis for the development of commercial biocontrol agents.

## Materials and Methods

### Rhizosphere Bacterial Isolates, Nematode Inoculum, and *P. notoginseng* Plants

The strains NS-2, NS-3, and GJ-7 were obtained from the rhizosphere soil of healthy *P. notoginseng* plants under the forest in Lancang city (Yunnan, China). Egg masses were obtained from *P. notoginseng* plants infected with the second-stage *Meloidogyne hapla* juveniles (J2s) grown in the greenhouses of the Yunnan Agricultural University. Eggs were obtained by washing the egg masses with 2% NaOCl solution, and the egg masses were incubated in sterile water at 28°C for 24 h to collect J2s (Hussey and Barker, 1973; Huang et al., 2016). In addition, the seedlings of *P. notoginseng* were obtained from the Daheqiao farm of Yunnan Agricultural University (103°16′49′′E, 25°31′2′′N) for pot experiments.

### Nematicidal Activity of Rhizosphere Bacterial Strains *in vitro*

First, the rhizosphere bacterial strains, NS-2, NS-3, and GJ-7, were cultured in 100 ml of LB liquid medium. The culture conditions were 30°C and 220 rpm for 48 h (Zhao et al., 2018). Then, the fermentation broth was collected by centrifuging the bacterial culture (8,000 rpm, 5 min) and filtering the supernatant with a 0.22-μm Millipore filter (Aalten and Gowen, 1998). The nematicidal activity of the rhizosphere bacterial strain was determined by a 24-well plate. Approximately 100 fresh J2s were contained in each well of a 24-well plate and treated with 1 ml of 100%, 50%, and 10% fermentation, 10% LB medium, sterilized water, and abamectin (1 μg/ml). The nematode mortality rate was recorded after 24-well plates were cultured at 28°C for 24 h. When the bodies of nematodes were straight and did not move upon stimulation with 0.5 M NaOH, the nematode was considered as dead (Harada and Yoshiga, 2015). The test was independently repeated three times, and each treatment had four replicates. The J2 mortality rate was calculated as follows: mortality rate = (the number of dead J2s/total J2s) × 100%.

### Inhibition of Egg Hatch by Rhizosphere Bacterial Strains *in vitro*

Similar to the J2 mortality test described above, the effects of the strains NS-2, NS-3, and GJ-7 on egg hatching were tested in 24-well plates. One milliliter of 100%, 50%, and 10% fermentation broth, 10% LB medium, LB medium, and sterilized water were mixed with 100 eggs in separate wells. The 24-well plates were incubated in a chamber at 28°C for 72 h, and the number of J2s hatched in each well was recorded (Mendoza et al., 2008; Saikia et al., 2013). The egg hatching rate was determined as follows: hatching rate = (the number of hatched J2s/total eggs) × 100%.

### Potential of Plant Rhizosphere Bacteria to Control *M. hapla* and the Effect on *P. notoginseng* Growth

Three experiments were designed for testing the potential of the strains NS-2, NS-3, and GJ-7 on biocontrol of the root-knot nematode and *P. notoginseng* growth promotion under greenhouse at Yunnan Agricultural University, China. *P. notoginseng* seedlings at the two-leaf stage were transplanted from the Daheqiao farm nursery land to individual pots filled with 200 g of sterilized soil. Then, the *P. notoginseng* seedlings were maintained at 25–28°C in a greenhouse. Treatments are as follows: (1) sterile water control, (2) LB medium control, (3) 1/1,000 dilution of abamectin control, (4) NS-2 fermentation broth, (5) NS-3 fermentation broth, and (6) GJ-7 fermentation broth. In Experiment 1, *P. notoginseng* plants were pre-inoculated with 10 ml of each of the three rhizosphere bacterial strains’ fermentation broth. Three days later, 1000 J2s of *M. hapla* in 5 ml of sterile water were inoculated into 2-cm-deep holes around each *P. notoginseng* stem. In Experiment 2, *P. notoginseng* plants were inoculated with 10 ml of rhizosphere bacteria fermentation broth and 1000 J2s at the same time. In Experiment 3, The 1000 J2s were inoculated into the *P. notoginseng* plants for 3 days, and then, 10 ml of bacterial fermentation broth was inoculated into each plant. Each treatment consisted of 10 pots and was repeated three times independently. The plant biomass values were measured after 30 days post-inoculation (dpi), including root length, stem length, stem fresh weight, and root fresh weight (Munif et al., 2013; Adam et al., 2014a). In addition, the level of nematode control was evaluated by detecting the number of galls on the roots of *P. notoginseng.*

### Analysis of *P. notoginseng* Root Colonization by Strains NS-2 and GJ-7

The strain NS-2 was tagged with the green fluorescent protein (GFP), and the strain GJ-7 was tagged with the red fluorescent protein (RFP) for colonization study. To generate the GFP-tagged strain NS-2 and RFP-tagged strain GJ-7, the pGFP4412 plasmid (Wangqi, Beijing) and the pRFP315 plasmid (stored in our lab) were introduced in the strains NS-2 and GJ-7 by electroporation at 1.8 kV, respectively. In order to ensure that the fluorescently labeled strains can be used for the colonization and migration experiment tracking of the roots of *P. notoginseng*, the growth curve of fluorescently labeled strains and wild-type (WT) strains was measured.

To quantify the amount of strains NS-2 and GJ-7 in *P. notoginseng* roots, the population of fluorescently labeled strains in *P. notoginseng* root was determined at 1, 3, 5, 7, 15, and 30 days post-inoculation (dpi). First, 10 ml of the suspension (1.0 × 10^7^ cfu/mL) was watered on *P. notoginseng* root. Three *P. notoginseng* plants were carefully taken out from the soil randomly each dpi. For the collection of roots samples, the *P. notoginseng* seedlings were delicately uprooted from the soil, and all loosely adhering bulk soil was removed, and 1 g of roots was put into 9 ml sterile water and vibrated on the oscillator for 30 min. The root samples were serially diluted and plated onto LB medium with an appropriate antibiotic. After culturing at 30°C for 2 days, the bacterial colonies were examined and counted from the roots.

### Live-Cell Imaging of *P. notoginseng* Roots Colonized by NS-2-GFP and GJ-7-RFP

In order to visually observe the colonization of the antagonistic strain in the root of *P. notoginseng*, the *P. notoginseng* seedlings were transferred to containers with 200 ml of 1/2 Hoagland culture medium and quartz sand. Then, they were inoculated with 20 ml of the NS-2-GFP or GJ-7-RFP overnight culture grown at 30°C in liquid LB supplemented with an appropriate antibiotic. Seedlings were incubated at 25°C for 5 days. Afterward, roots were washed with distilled water to eliminate non-adhered bacteria, and the root system was examined with a laser scanning confocal microscopy.

### Dynamic Expression of Disease Resistance-Related Genes of *P. notoginseng* Root

Two-leaf stage *P. notoginseng* seedlings were transferred to individual pots filled with 200 g of sterilized soil and maintained in a greenhouse as previously described. One week after transplanting, 10 ml of the GJ-7 and NS-2 cultured strains (1.0 × 10^7^ cfu/mL) were watered on *P. notoginseng* roots as described previously. Three root samples were prepared at 0-, 12-, 24-, 48, and 72-h time points and stored at −80°C after quick freezing with liquid nitrogen. To perform gene expression analysis, the total RNA was extracted from root samples using the Pure Plant Kit (Omega), and cDNA was synthesized using the PrimeScript RT Reagent Kit with gDNA Eraser (Takara, Dalian). The expression of *PnACT2* (internal control), *PnPR1*, and *PnCHI1* genes was analyzed by a real-time PCR SYBR Green I reaction system using gene-specific primers, as described previously (Bai et al., 2018; Zhang et al., 2020). The expression level of each gene was normalized and related to that of *PnACT2*, which was quantified using the ^ΔΔ^Ct method (Beaubois et al., 2007).

### Statistical Analysis

All data were recorded and organized in Microsoft Office Excel (2010). Statistical analysis was carried out using one-way analysis of variance (ANOVA) with Duncan’s multiple range test (*P* < 0.05) and Tukey’s multiple comparison test (*P* < 0.001) in DPS.

## Results

### Evaluation of Nematicidal Activity of Strains NS-2, NS-3, and GJ-7 *in vitro*

**FIGURE 1:**
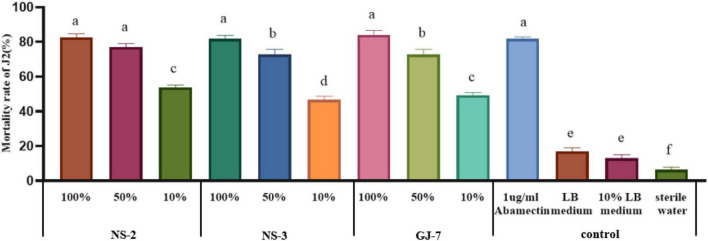
The mortality rate of the second-stage juveniles (J2s) of *Meloidogyne hapla* induced by *Bacillus cereus* strain NS-2, *Lysinibacillus strain* NS-3, and *Bacillus velezensis* GJ-7 cell-free supernatant *in vitro*. Abamectin (1 μg/ml), a strong biological nematicide, was used as a positive control, while LB medium, 10% LB medium, and sterilized water were used as negative controls. Data were analyzed by one-way ANOVA with Duncan’s multiple range test, and error bars represent the standard error of three biological and ten technical replicates (*n* = 30). Different lowercase letters indicate significant differences among treatments (*P* < 0.05).

The mortality rate of J2s in the LB medium, 10% LB medium, and sterilized water was 17%, 13%, and 6.3% at 24 h, respectively (Figure 1). All fermentation broth treatments indicated significantly higher nematicidal activity against *M. hapla* J2s than negative controls (10% LB medium, LB medium, and sterilized water) at 24 h. Among them, the mortality rate of J2s in 100% fermentation broth of NS-2 was 82.6%, NS-3 was 81.6%, and GJ-7 was 84%. The mortality rate of J2s in 50% fermentation broth of NS-2 was 77%, NS-3 was 72.6%, and GJ-7 was 72.7%. The mortality rate of J2s in 10% fermentation broth of NS-2 was 53.66%, NS-3 was 46.67%, and GJ-7 was 49.33% (Figure 1). Although the mortality rate of J2s in the abamectin (positive control) was 81.66% at 24 h, the 100% and 50% fermentation broth of three rhizosphere bacterial strains had the same effect (Figure 1). The results of the nematicidal activity test *in vitro* showed that the fermentation broth of three rhizosphere bacteria displayed strong nematicidal abilities (Figure 1).

### Effect of Strains NS-2, NS-3, and GJ-7 on the Hatching of *M. hapla* Eggs

**FIGURE 2:**
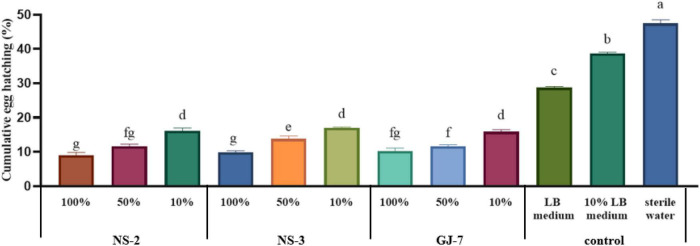
The egg hatching rate of *Meloidogyne hapla* exposed to *Bacillus cereus* strain NS-2, *Lysinibacillus* strain NS-3, and *Bacillus velezensis* GJ-7 cell-free supernatant *in vitro*. LB medium, 10% LB medium, and sterilized water were used as negative controls. Data were analyzed by one-way ANOVA with Duncan’s multiple range test, and error bars represent standard error of three biological and ten technical replicates (*n* = 30). Different lowercase letters indicate significant differences of 72 h among treatments (*P* < 0.05).

At 72 h, at the highest concentration tested, the egg hatching rate was the lowest when treated with strain NS-2 fermentation (9.01%), followed by the NS-3 fermentation (9.86%) and GJ-7 fermentation (10.25%). At a concentration of 10 times diluted, the egg hatching rate of all fermentation treatments still was significantly lower than the LB (28.73%), 10% LB (38.73%), and sterilized water control (47.47%) (Figure 2). The egg hatching rate in 10% fermentation broth of NS-2 was 16.09%, NS-3 was 17.08%, and GJ-7 was 15.91% (Figure 2). The results *in vitro* showed that the fermentation broth of three rhizosphere bacterial strains could significantly inhibit the hatching of eggs.

### Effect of Rhizosphere Bacteria in Biocontrol of *M. hapla* and Plant Growth Promotion in *P. notoginseng*

**FIGURE 3:**
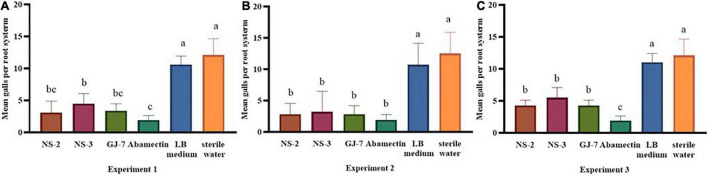
The rhizosphere bacterial strains NS-2, NS-3, and GJ-7 in biological control potential of *Panax notoginseng* plants infected with *Meloidogyne hapla* in three treatments of pot experiment. Means in each column followed by the same letter do not differ significantly according to Duncan’s multiple range test at *P* ≤ 0.05.

The effect of rhizosphere bacteria fermentation broth in biocontrol of *M. hapla* and plant growth promotion was verified in a pot experiment. The results indicated that inoculation of the NS-2, NS-3, and GJ-7 strains in *P. notoginseng* roots in different experiments significantly reduced the number of galls compared with the negative control (sterile water and LB medium) (Figure 3). In the pre-inoculation of rhizosphere bacteria or rhizosphere bacteria and *M. hapla* that were inoculated at the same time treatments, abamectin (positive control) showed higher efficacies in reducing the number of galls, but there was no significant difference with NS-2 and GJ-7 treatments (Figures 3A,B). Compared with the water control, abamectin treatment reduced the number of galls by 85%, and pre-inoculation of NS-2, NS-3, and GJ-7 treatments reduced the number of galls by 74.3%, 63.3%, and 72.5%, respectively. When rhizosphere bacteria and *M. hapla* were inoculated at the same time treatment, the number of galls reduced by 77.6%, 74%, and 77.6%, respectively. Although, the pre-inoculation with *M. hapla* did not have a solid effect on the three bacteria isolates on the reduction of the galls compared with abamectin control (Figure 3C). However, the number of galls still decreased by 64%, 54.5%, and 64.4%, respectively. In addition, all treatments with NS-2 and GJ-7 exhibited higher efficacies in reducing the number of galls, followed by NS-3. These results suggest that rhizosphere bacteria have protective and therapeutic effects. In contrast, the protective effect is stronger, which may be related to the predetermined colonization of rhizosphere bacteria. The growth parameters presented in Supplementary Table 1 revealed that the three rhizosphere bacterial isolates have different effects on plant growth. In all treatments, NS-2 and GJ-7 enhanced root length and stem length, but compared with the control, the root fresh weight and stem fresh weight are not significant (Supplementary Table 1).

### Construction of GFP-Tagged Strain NS-2 and RFP-Tagged Strain GJ-7

**FIGURE 4:**
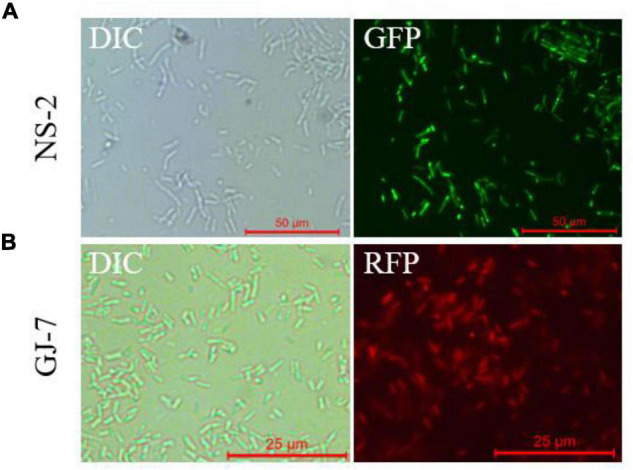
Fluorescence observation of NS-2 and GJ-7 under microscopy. DIC, differential interference contrast field; RFP, red fluorescent field; and GFP, green fluorescent field. Representative fluorescent micrograph of **(A)** NS-2 and **(B)** GJ-7 under a fluorescent microscope.

**FIGURE 5:**
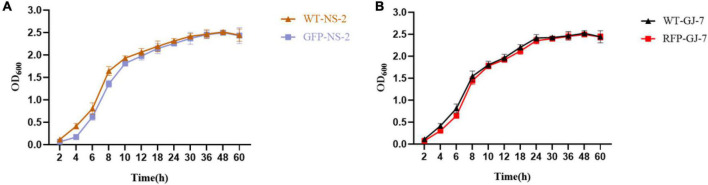
The growth rate of the tagged strain compared with the wild-type strain. OD_600_ measures were used to determine bacterial growth rate. **(A)** Growth rate of the strain NS-2. **(B)** Growth rate of the strain GJ-7. Three replicates were included.

We successfully transferred the green fluorescent protein plasmid pGFP4412 and the red fluorescent protein plasmid pRFP315 to the strains NS-2 and GJ-7, respectively. The transformed strains were observed under a fluorescence microscope, and the results showed that the plasmid has been successfully transformed into the NS-2 and GJ-7 strains and could express the fluorescent normally (Figure 4). In addition, the growth curve of the GFP-tagged strain NS-2, RFP-tagged strain GJ-7, and WT strains was measured under the same inoculation and culture conditions. The results showed that the growth of GFP-NS-2 and RFP-GJ-7 was consistent with WT strains (Figure 5), indicating that the plasmids, pGFP4412 and pRFP315, had no significant effect on the growth of NS-2 and GJ-7 strains, and the tagged strains can be used for experiment tracing colonization and migration in plants.

### Colonization Assay of Strains NS-2 and GJ-7 in the *P. notoginseng* Roots

**FIGURE 6:**
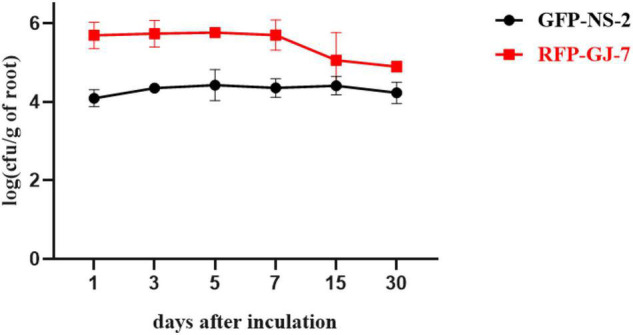
Population of GFP-NS-2 and RFP-GJ-7 on *Panax notoginseng* roots. Population is expressed in log (cfu/g) of roots (y-axis) and days (x-axis).

The population of GFP-tagged strain NS-2 and RFP-tagged strain GJ-7 on *P. notoginseng* roots was monitored at 1, 3, 5, 7, 15, and 30 days after inoculation, as described in Materials and Methods. GFP-NS-2 and RFP-GJ-7 can rapidly proliferate in large quantities within 5 dpi on the *P. notoginseng* roots. The population of GFP-NS-2 and RFP-GJ-7 in the roots was stable, with ≥ 10^3^ cfu/g of fresh weight until 30 dpi (Figure 6). In addition, RFP-GJ-7 was found to have the strongest colonization ability, and the maximum density of cells was 5.63 × 10^5^ cfu/g of fresh weight at 5 dpi on the *P. notoginseng* roots; overall colonization was significantly higher than GFP-NS-2 (*P* ≤ 0.05).

To explore the colonization patterns of GFP-NS-2 and RFP-GJ-7 in *P. notoginseng* roots, we used the laser confocal microscope (LCM) to observe the roots of *P. notoginseng* inoculated with the GFP-NS-2 and RFP-GJ-7 strains for 5 days, respectively.

**FIGURE 7:**
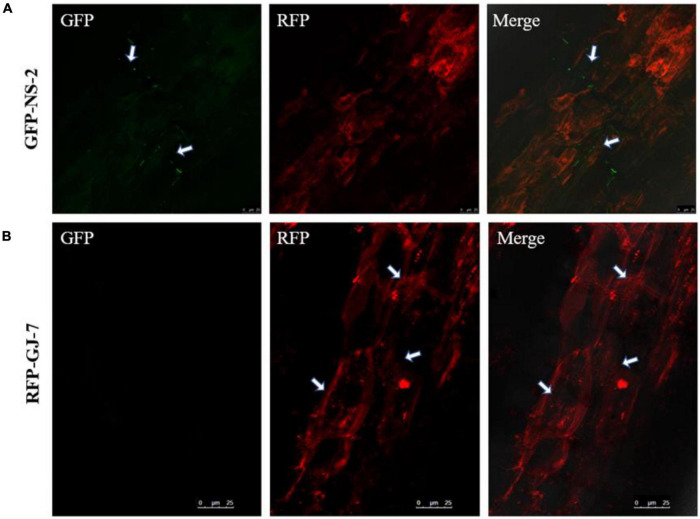
Colonization observation of the strains NS-2 and GJ-7 on *Panax notoginseng* roots. **(A)** Images of GFP-tagged strain NS-2-treated roots in the dark field (GFP), the dark field (RFP), and the superposition field (merged). **(B)** Images of RFP-tagged strain GJ-7-treated roots in the dark field (GFP), the dark field (RFP), and the superposition field (merged).

The results indicate that *P. notoginseng* roots were colonized by GFP-NS-2 and RFP-GJ-7, and GFP-NS-2 and RFP-GJ-7 were observed on root epidermal cells at 5 days after inoculation (DAI) (Figure 7).

### Strains NS-2 and GJ-7 Changing Defense-Responsive Gene Expression

**FIGURE 8:**
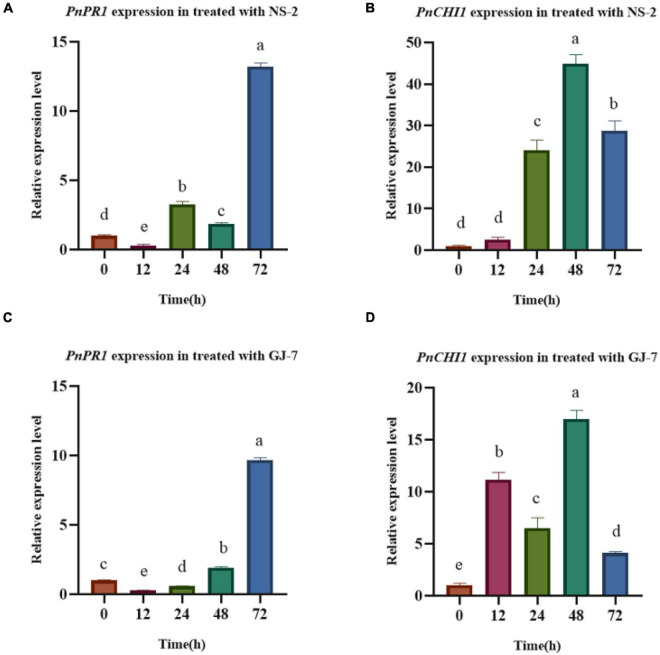
Analysis of defense response in *Panax notoginseng* roots treated with the strains NS-2 and GJ-7. **(A,B)** Relative expression level of *PnPR1* **(A)** and *PnCHI1* **(B)** in *P. notoginseng* roots treated with strain NS-2. **(C,D)** Relative expression level of *PnPR1* **(C)** and *PnCHI1* **(D)** in *P. notoginseng* treated with the strain GJ-7. Expression levels were normalized relative to the control at the 0 h time point. Data were analyzed by one-way ANOVA with Duncan’s multiple range test, and error bars represent standard error of three biological and three technical replicates (*n* = 9). Different lowercase letters indicated significant differences among treatments (*P* < 0.05).

The inoculation treatment with the NS-2 and GJ-7 strains upregulated the expression in *P. notoginseng* root of *PnPR1* and *PnCHI1* genes. The *PR1* gene-encoding proteins are involved in the SA signaling pathway. In addition, chitinase (CHI) is also an important part of the disease resistance and defense response system. In the inoculation treatment with NS-2 strain, the expression of *PnPR1* and *PnCHI1* gene was significantly upregulated compared with the control (0 h) after 24 h (Figures 8A,B). In the inoculation treatment with GJ-7 strain, the expression of *PnPR1* gene was lower than that of the control within 24 h of inoculation, but its expression has a trend of continuous growth. After 48 h, the *PnPR1* gene expression level exceeds that of the control. The expression level of *PnCHI1* gene was higher than the control in all time periods, reaching a peak at 48 h (Figures 8C,D). These results indicated that the strains NS-2 and GJ-7 activate the defense signaling pathways in *P. notoginseng* roots.

## Discussion

Antagonistic bacteria are generally considered as effective microorganisms for the biocontrol of root-knot nematode (Adam et al., 2014b; Xiong et al., 2015) as well as plant growth-promoting bacteria (Vetrivelkalai et al., 2010). However, few studies have reported the possibility of *B. cereus* and *B. velezensis* as rhizosphere bacteria to serve as a biocontrol of *M. hapla*. In this study, the *B. cereus* NS-2, *Lysinibacillus* NS-3, and *B. velezensis* GJ-7 from the rhizosphere of *P. notoginseng* plants showed strong nematicidal activity to *M. hapla* J2s and suppressed the hatching of eggs *in vitro*. These results indicated that three rhizosphere bacteria may kill *M. hapla* J2s and its eggs before infecting *P. notoginseng* plants. In addition, the results of the pot experiment showed that *B. cereus* NS-2 and *B. velezensis* GJ-7 could effectively control *M. hapla* and promote plant growth. These data suggest that the strains NS-2 and GJ-7 can be developed as biological nematicides to control *M. hapla*.

In addition, comparing the efficiency biocontrol of *M. hapla* under three experiments, infection by *M. hapla* was significantly reduced by rhizosphere bacteria pre-colonization. These results indicate that root colonization by rhizosphere bacteria is a prerequisite for the biological control of pathogens. As previously reported, some beneficial microorganisms can colonize the root surface to form biofilm so as to protect plants against pathogen infection (Davey and O’toole, 2000; O’Toole et al., 2000). Root colonization by bacteria could prevent nematode infection by inhibiting J2 hatching, motility, and viability (Mendoza et al., 2008; Terefe et al., 2009; Xiong et al., 2015). In this study, to confirm the colonization of *P. notoginseng* roots by the strains NS-2 and GJ-7, we generated the GFP-tagged strain NS-2 and RFP-tagged strain GJ-7. In our experiment, the strains NS-2 and GJ-7 occupied the ecological niche, with a rapidly growing population of up to 3.46 × 10^4^ and 3.53 × 10^5^ cfu/g of roots for 5 dpi, respectively. The population of two bacterial colonization was stabilized in *P. notoginseng* roots and maintained at 10^3^ cfu/g of roots in 30 days. The colonization of NS-2 and GJ-7 on the root surface of *P. notoginseng* was also observed under the CLSM. These data indicate that the strains NS-2 and GJ-7 have a large ability of establishment and persistence either on the *P. notoginseng* roots.

Previously, some studies reported that bacterial colonization of the root surface can enhance the host defense mechanism and reduce the invasion of RKNs (Xiang et al., 2017). Vos et al. (2013) suggested that bacteria might enhance the activity of biomolecules and enzymes related to plant defense against root-knot nematodes. The major defense mechanisms of plants were regulated through SA and JA signaling pathways, and the *PR1* gene was the marker of the signaling pathway (Shoresh et al., 2005; Islam et al., 2008; Pulla et al., 2010). The *PnPR1* gene is the only gene cloned in the PR1 family of *P. notoginseng* (Zhang et al., 2020). In this study, the expression of *PnPR1* gene was increased, indicating that the defense response of *P. notoginseng* to *M. hapla* mediated by the SA signaling pathway was activated. In addition, chitinase (CHI) is also an important part of the disease resistance and defense response system. Studies have shown that chitinase has the activity of inhibiting the growth of fungal hyphae (Bai et al., 2018). The increase in the expression of the *PnCHI1* gene (the chitinase gene in *P. notoginseng*) also indicates the activation of the defense response of *P. notoginseng.* Due to the limitation of reports on the defense-related genes of *P. notoginseng*, this study only proved the expression of two related genes. However, these data confirmed that the strains NS-2 and GJ-7 activate defense responses in *P. notoginseng* roots against *M. hapla*.

In summary, the *B. cereus* NS-2 and *B. velezensis* GJ-7 from the rhizosphere of *P. notoginseng* exhibited direct nematicidal and egg inhibition against *M. hapla* and formed a biofilm on the surface of *P. notoginseng* roots, which effectively prevented nematode invasion. In addition, the strains NS-2 and GJ-7 also stimulated defensive responses to enhance plant resistance against *M. hapla*. Taken together, our results revealed a possible anti-nematode mechanism of rhizosphere bacteria from *P. notoginseng*. *B. cereus* NS-2 and *B. velezensis* GJ-7 with nematicidal activity help *Panax notoginseng* against root-knot nematodes through rapid colonization and mediated resistance.

## Data Availability Statement

The raw data supporting the conclusions of this article will be made available by the authors, without undue reservation.

## Author Contributions

WW conceived, designed, and performed the experiment, analyzed the data, and wrote the manuscript. YW conceived and designed the experiment and prepared the manuscript. JW and ZW analyzed the data. LG and SZ participated in the revision of the manuscript. XH and YZ supervised the research and provided funding support. All authors contributed to the article and approved the submitted version.

## Conflict of Interest

The authors declare that the research was conducted in the absence of any commercial or financial relationships that could be construed as a potential conflict of interest.

## Publisher’s Note

All claims expressed in this article are solely those of the authors and do not necessarily represent those of their affiliated organizations, or those of the publisher, the editors and the reviewers. Any product that may be evaluated in this article, or claim that may be made by its manufacturer, is not guaranteed or endorsed by the publisher.
