# Supplementary material for: Rhizosphere Bacteria From Panax notoginseng Against Meloidogyne hapla by Rapid Colonization and Mediated Resistance
Source: Front Microbiol. 2022 Apr 28;13:877082. doi: 10.3389/fmicb.2022.877082 (PMC9096944; doi:10.3389/fmicb.2022.877082)
Supplement: Supplementary file 1 [file Table_1.docx]

|  | **Treatment** | **Shoot length (cm)** | **Stem length**  **(cm)** | **Root fresh weight (g)** | **Stem fresh weight (g)** |
| --- | --- | --- | --- | --- | --- |
| **Experiment 1** | Water | 9.61±2.53 | 12.22±1.84 | 0.61±0.23 | 0.58±0.11 |
|  | LB medium | 9.86±1.72 | 12.45±1.01 | 0.61±0.09 | 0.59±0.05 |
|  | Abamectin | 9.96±1.63 | 12.8±1.39 | 0.61±0.12 | 0.59±0.05 |
|  | NS-2 | 11.05±1.60 | 13.12±1.37 | 0.60±0.18 | 0.60±0.16 |
|  | NS-3 | 9.81±1.19 | 12.3±0.70 | 0.56±0.09 | 0.54±0.07 |
|  | GJ-7 | 11.05±1.61 | 12.75±1.57 | 0.61±0.15 | 0.59±0.06 |
| **Experiment 2** | Water | 7.86±1.42 | 11.5±1.29 | 0.44±0.10 | 0.53±0.07 |
|  | LB medium | 8.25±1.63 | 12.70±1.84 | 0.454±0.12 | 0.56±0.16 |
|  | Abamectin | 8.45±1.36 | 11.75±1.35 | 0.44±0.11 | 0.57±0.16 |
|  | NS-2 | 7.95±2.62 | 12.35±2.14 | 0.42±0.07 | 0.53±0.09 |
|  | NS-3 | 7.05±1.70 | 12.65±1.65 | 0.41±0.05 | 0.57±0.11 |
|  | GJ-7 | 9.4±2.07 | 14.4±1.98 | 0.53±0.12 | 0.71±0.21 |
| **Experiment 3** | Water | 9.61±2.53 | 12.22±2.14 | 0.61±0.23 | 0.58±0.11 |
|  | LB medium | 9.81±1.82 | 12.55±1.42 | 0.65±0.12 | 0.62±0.08 |
|  | Abamectin | 9.75±1.34 | 13.05±1.23 | 0.63±0.07 | 0.60±0.07 |
|  | NS-2 | 10.30±2.07 | 13.15±1.33 | 0.61±0.15 | 0.63±0.08 |
|  | NS-3 | 9.5±1.10 | 12.4±0.84 | 0.58±0.09 | 0.59±0.1 |
|  | GJ-7 | 11.2±1.42 | 14.05±1.27 | 0.75±0.20 | 0.62±0.11 |

Supplementary Table. 1. The rhizosphere bacteria NS-2, NS-3 and GJ-7 effect on the growth parameters of *panax notoginseng* plants infected with *M. hapla* in three treatments of pot experiment. Data are represented as the mean± standard deviation (n=10).
